# Supplementary material for: Adaptive traits of cysts of the snow alga Sanguina nivaloides unveiled by 3D subcellular imaging
Source: Nat Commun. 2023 Nov 18;14:7500. doi: 10.1038/s41467-023-43030-7 (PMC10657455; doi:10.1038/s41467-023-43030-7)
Supplement: Supplementary file 1 — Supplementary Information [file 41467_2023_43030_MOESM1_ESM.pdf]

Adaptive traits of cysts of the snow alga *Sanguina nivaloides*

unveiled by 3D subcellular imaging

Jade A. Ezzedine, Clarisse Uwizeye, Grégory Si Larbi, Gaelle Villain, Mathilde Louwagie, Marion Schilling, Pascal Hagenmuller, Benoît Gallet, Adeline Stewart, Dimitris Petroutsos, Fabienne Devime, Pascal Salze, Lucie Liger, Juliette Jouhet, Marie Dumont, Stéphane Ravanel, Alberto Amato, Jean-Gabriel Valay, Pierre-Henri Jouneau, Denis Falconet, Eric Maréchal

Supplementary Material

Supplementary Figures

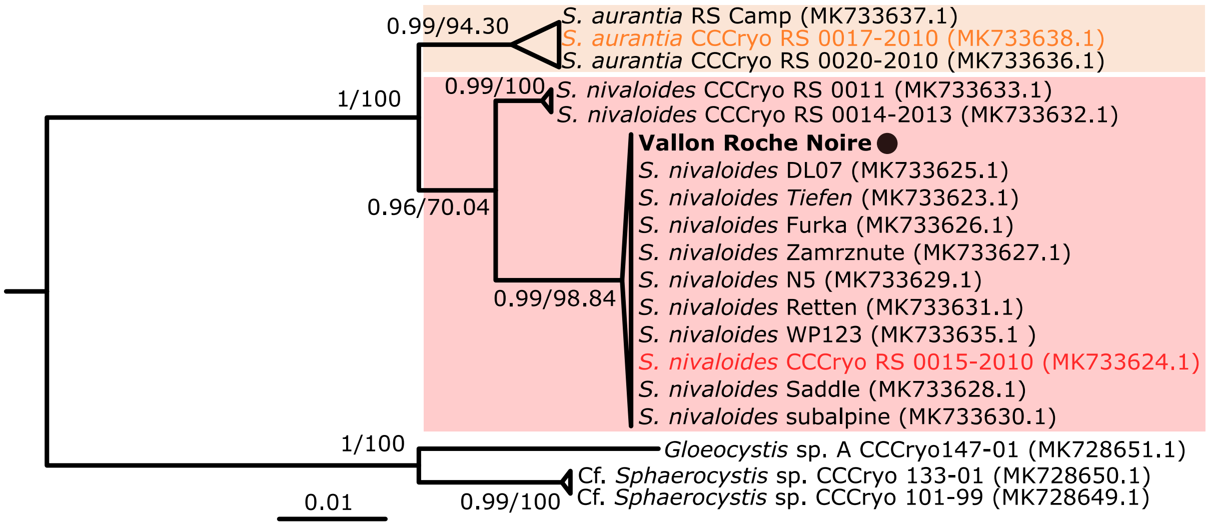

Supplementary Figure 1: **Phylogenetic analysis of Vallon Roche Noire algal blooms, based on rbcL sequence.** The rbcL gene-based Bayesian phylogenetic tree was built using a data set of 19 aligned sequences with 616 positions of *Sanguina* using MUSCLE via MEGAX. Best model was calculated with ModelTest-NG v0.1.7. The best model was given as follows: GTR+G (0.1883); base frequencies: A 0.2971, C 0.1859, G0.1967, T 0.3203 and rate matrix A-C 0.2432, A-G 1.4545, A-U 2.6253, C-G 0.0899, C-U 5.2740, G-U 1.0000. The tree shown was inferred with Bayesian method using MrBayes v3.2.7a and maximum likelihood method using PhyML v3.3.2. Posterior probabilities (PP) followed by bootstraps (BS) values (in percentage) are added to the left of nodes. The tree is supported with PP >95 and BS >70. *Gloeocystis* sp., and two *Sphaerocystis* sp. were used as outgroup to root the tree. *Sanguina aurantia* sequences are highlighted in yellow and *S. nivaloides* in red. Vallon Roche Noire sequence is indicated with a black dot. Accession numbers, strain or field sample codes are shown after each species name.

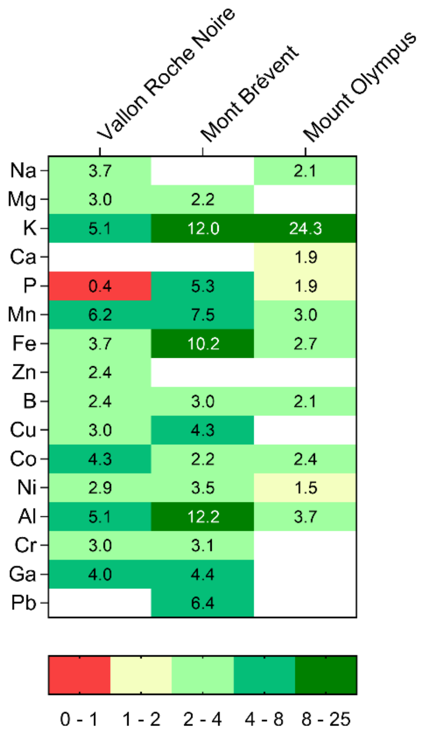

Supplementary Figure 2. Variations of essential element contents in liquid water from red *vs* white snowfields. Elemental composition of red snow and white snow samples were analyzed in three sites. One corresponds to Vallon Roche Noire (bloom 3), where most analyses presented where performed, one in Mont Brévent (bloom 5), in the same mountain range of the French Alps. A site at Mount Olympus, Greece, was also analyzed, corresponding to a mountain range with a different rock system, being calcareous. The presence of *S. nivaloides* as the major algal species was verified in all sites. ICPMS analyses were performed in 5 replicates and the ratio between elemental concentration in red and white snow was calculated from mean values. The heatmap displays ratio values for essential and non-essential elements at the three sampling sites.

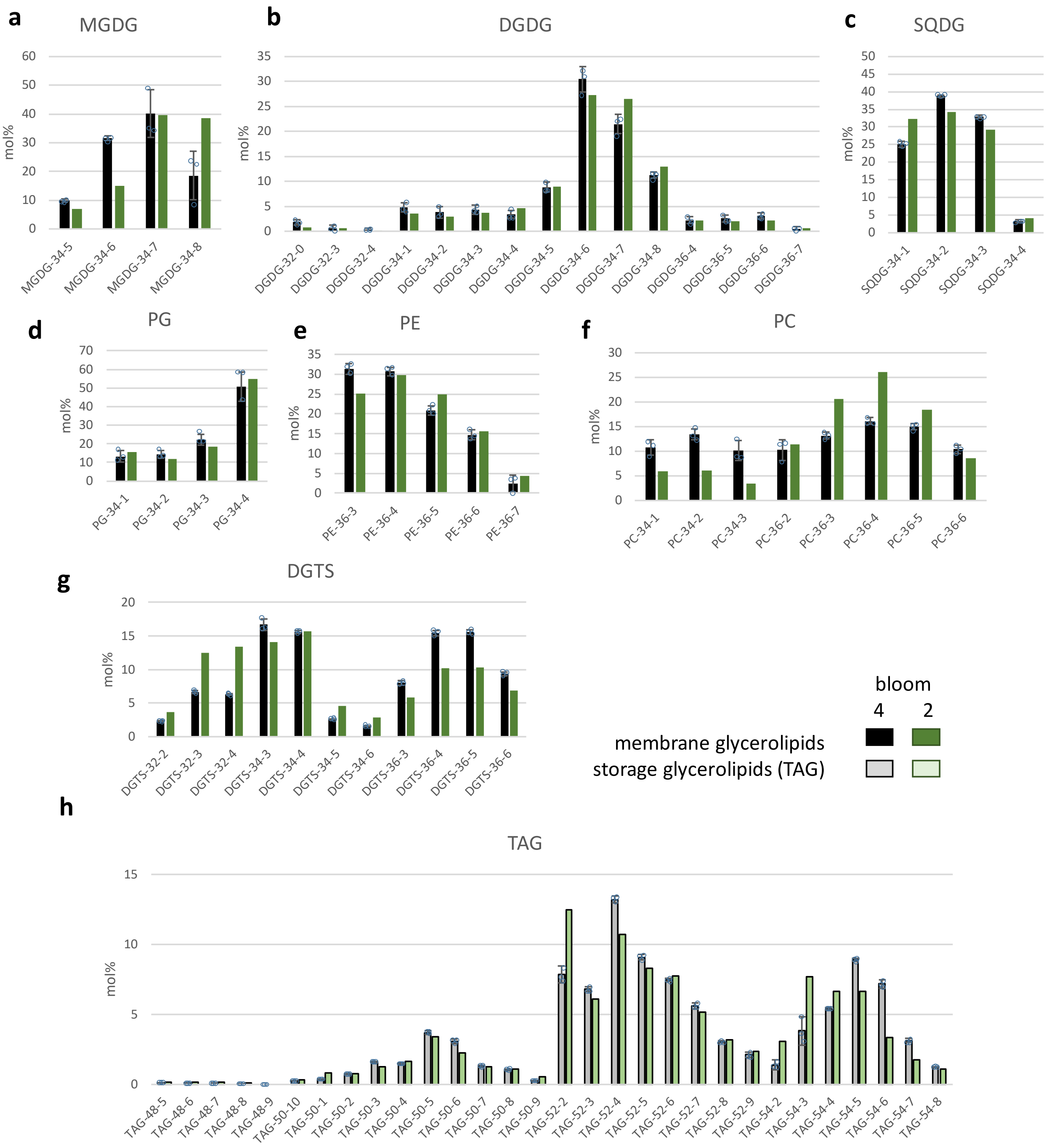

Supplementary Figure 3. Molecular species of glycerolipids in *S. nivaloides* cysts collected in two independent blooms of the Lautaret area. Each lipid class was analyzed by LC-MS as described in Methods. Molecular species are expressed in total number of carbons and of double bonds, summed in the two acyls at the *sn*-1/*sn*-2 positions of the glycerol backbone in membrane glycerolipids **(a-g)** and in the three acyls at the *sn*-1/*sn*-2/*sn*-3 position in TAG **(h)**. Relative proportions are expressed in mol%. Profiles obtained from bloom 2 (in a single replicate, due to quantity limitation) and bloom 4 (in triplicate) show high level of consistency. DGDG, digalactosyldiacylglycerol; DGTS, diacylglyceryl-trimethylhomoserine; MGDG, monogalactosyldiacylglycerol; PC, phosphatidylcholine; PE, phosphatidylethanolamine; PG, phosphatidylglycerol; SQDG, sulfoquinovosyldiacylglycerol; TAG, triacylglycerol.

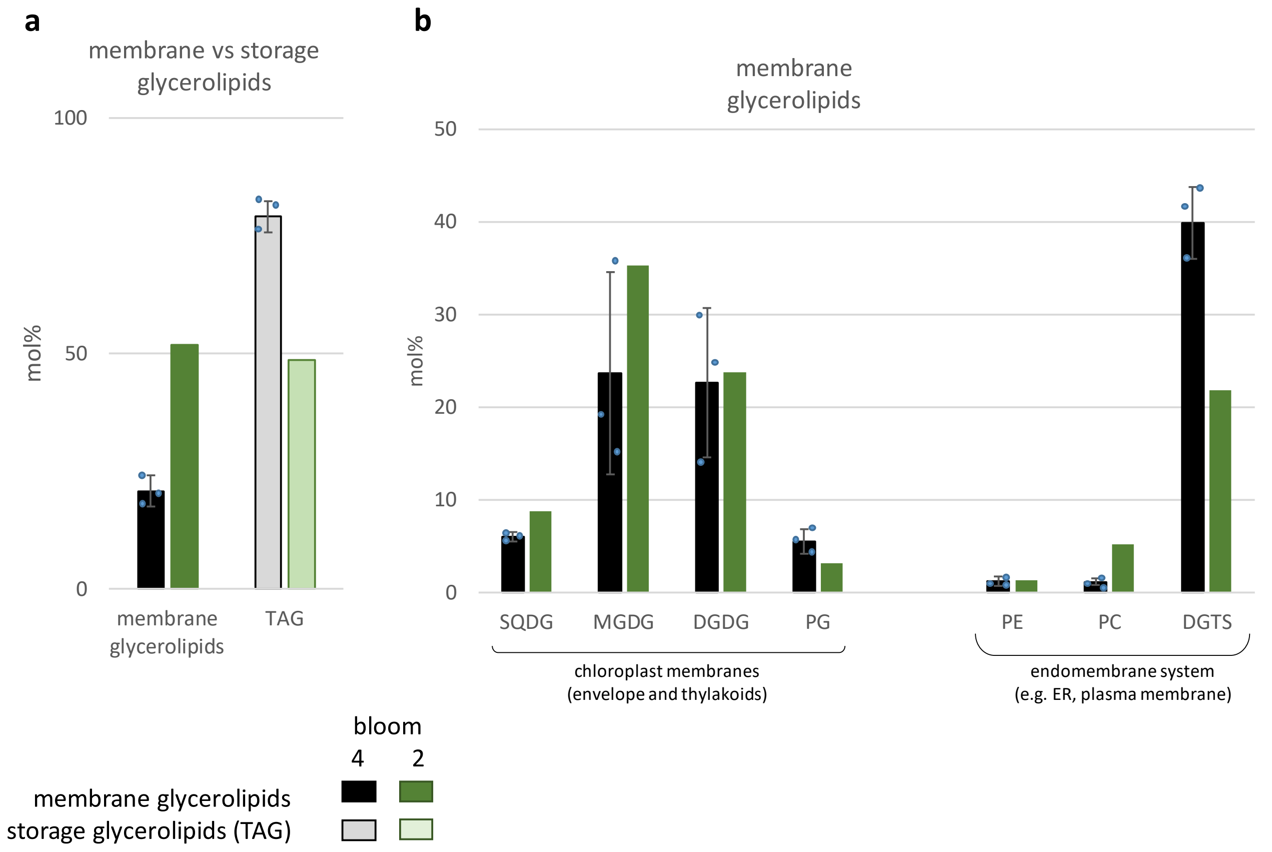

Supplementary Figure 4. Comparison of glycerolipid profiles in *S. nivaloides* cysts collected in two independent blooms of the Lautaret area. (a) Relative proportions of membrane and storage glycerolipids. Relative proportions are expressed in mol% of total glycerolipids. **(b) Relative proportions of membrane glycerolipid classes.** Relative proportions are expressed in mol% of membrane glycerolipids. Profiles obtained from bloom 2 (in a single replicate, due to quantity limitation) and bloom 4 (in triplicate) show high level of consistency. In both blooms, the three lipidomic signatures of a phosphate limitation are visible, i.e. an accumulation of TAG, and SQDG-to-PG and DGTS-to-PC replacements. DGDG, digalactosyldiacylglycerol; DGTS, diacylglyceryl-trimethylhomoserine; MGDG, monogalactosyldiacylglycerol; PC, phosphatidylcholine; PE, phosphatidylethanolamine; PG, phosphatidylglycerol; SQDG, sulfoquinovosyldiacylglycerol; TAG, triacylglycerol.

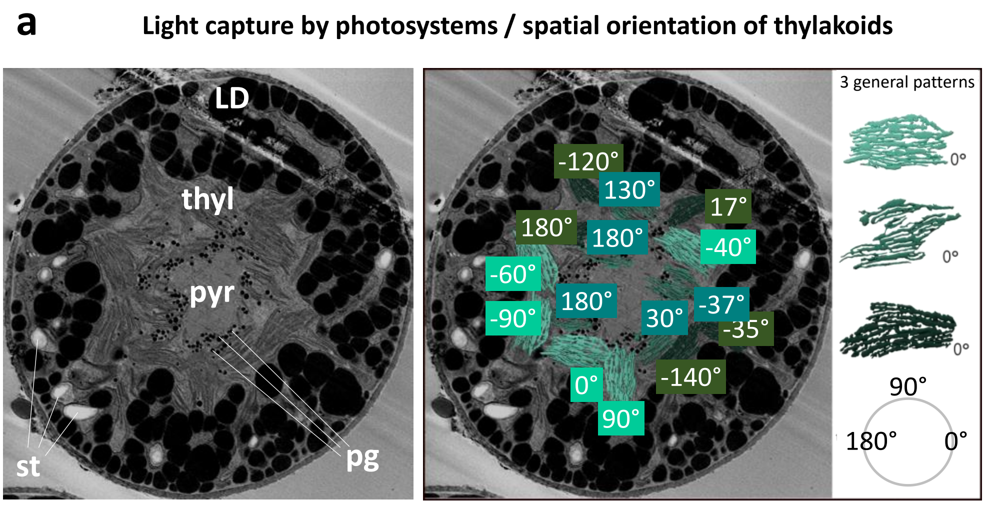

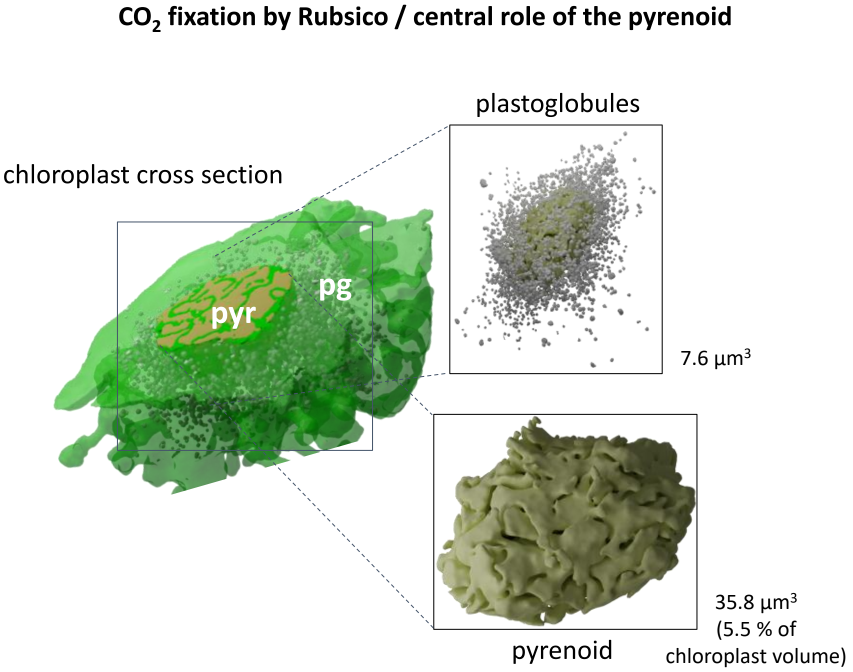

Supplementary Figure 5. Chloroplast sub-organellar architecture. (a) Thylakoid orientation allowing the capture of incident light coming from all directions. Based on a piecewise segmentation of membranes, a comparison of thylakoid pile patterns was established for the XY orientation of the section. Three general thylakoid pile patterns based on the organization of parallel and or branched thylakoids, are illustrated in green color scale. The orientation of each pattern in the cross section illustrates the capacity to capture a perpendicular incident light, coming from all directions. Multiple orientations of thylakoids were observed in all cysts imaged in this work. This analysis was performed on a single cell. **(b) 3D reconstruction of the pyrenoid and plastoglobules.** The central sponge-like pyrenoid is shown in light brown. LD, lipid droplet; thyl, thylakoids; pyr, pyrenoid; pg, plastoglobules; st, starch. This reconstruction was performed on a single cell.

Supplementary Tables

Supplementary Table 1. Relative abundance (%) of membrane glycerolipid species (as a function of total species concentration within each class) in a total lipid extract from *S. nivaloides* bloom 2. Lipid classes were monogalactosyldiacylglycerol (MGDG), digalactosyldiacylglycerol (DGDG), sulfoquinovosyldiacylglycerol (SQDG), phosphatidylcholine (PC) and diacylglyceryl-trimethylhomoserine (DGTS). The carbon:double bonds column refers to the total number of carbon atoms and double bonds of acyl groups attached to the diglycerid moiety. The *sn*-1/*sn*-2 molecular species refer to the dominant fatty acid combinations at the *sn*-1 and *sn*-2 position of the glycerol backbone as detected in MS spectra (see Materials and Methods). When the position of acyl groups cannot be assessed without ambiguity, the sb-1/sn-2 position os indicated with "-". * and ** indicate major molecular species reported previously in *Chlamydomonas reinhardtii* and *Chlorella* sp., respectively. Molecular species not found in *C. reinhardtii* and *Chlorella* sp. are shown in red.

Lipid class	carbon:double bonds	sn-1/sn-2 molecular species	
MGDG	34:8	**18:4/16:4**	38.37%
MGDG	34:7*,**	18:3/16:4 + (18:4/16:3)	31.93%
MGDG	34:6	18:2/16:4 + 18:3/16:3	10.18%
MGDG	34:5	18:1/16:4 + 18:2/16:3 + (18:3/16:2)	4.14%
MGDG ox	34:8 ox	**18:4/16:4 ox** + **18:4 ox/16:4**	6.24%
MGDG ox	34:7 ox	18:3-16:4 ox + 18:3 ox/16:4	6.09%
MGDG ox	34:6 ox	18:3-16:3ox + 18:3 ox-16:3	3.05%
DGDG	34:7	18:3/16:4	29.65%
DGDG	34:6**	18:3/16:3** + 18:2-16:4	27.71%
DGDG	34:5	18:2/16:3 + 18:3/16:2 + 18:1/16:4	8.48%
DGDG	34:1	18:1/16:0	6.28%
DGDG	34:3*	18:3/16:0* + (18:2/16:1 + 18:1/16:2)	5.64%
DGDG	34:2	18:2/16:0 + 18:1/16:1	5.32%
DGDG	34:4	18:1/16:3 + 18:2/16:2 + 18:3/16:1	4.94%
DGDG	36:5	18:3-18:2 + (20:1/16:4 + **18:4/18:1**)	2.85%
DGDG	36:4	18:2/18:2 + 18:1-18:3	2.53%
DGDG	36:6	18:3/18:3	1.35%
DGDG	34:8	**18:4/16:4**	1.22%
DGDG	36:7	**18:4/18:3**	1.19%
SQDG	34:1	18:1/16:0	37.47%
SQDG	34:3**	18:3/16:0	31.18%
SQDG	34:2	18:2/16:0	20.85%
SQDG	34:0	**18:0/16:0**	6.29%
SQDG	34:4	**18:4/16:0**	4.20%
PC	36:2	18:1/18:1	21.88%
PC	36:5**	18:2-18:3	19.56%
PC	36:4	18:2/18:2	13.66%
PC	36:3	18:2-18:1	16.57%
PC	34:1	18:1-16:0	7.40%
PC	34:3	18:3-16:0	7.05%
PC	34:2	18:2-16:0	7.02%
PC	36:6**	18:3/18:3	6.82%
DGTS	32:4	16:4/16:0	20.68%
DGTS	34:4	18:4/16:0 + (16:3-18:1)	19.39%
DGTS	36:5	18:4/18:1 + 18:3/18:2	11.48%
DGTS	36:6	18:3/18:3 +18:4/18:2	9.90%
DGTS	32:3	16:3/16:0 + (14:0-18:3)	9.15%
DGTS	34:3*	18:3/16:0	7.27%
DGTS	34:5	16:4/18:1 + 16:3/18:2 + (16:2/18:3 + 16:1-18:4)	6.56%
DGTS	36:4	18:3/18:1 + 18:2/18:2 + (18:4/18:0)	6.33%
DGTS	34:6	16:4/18:2 + 16:3/18:3	6.26%
DGTS	36:3	18:2/18:1 + (18:3/18:0)	2.92%

Supplementary Table 2. Relative abundance (%) of triacylglycerol (TAG) species (as a function of total TAG species) in a total lipid extract from a *S. nivaloides* bloom. The carbon:double bonds column refers to the total number of carbon atoms and double bonds of acyl groups attached to the diglycerid moiety. The *sn*-1/*sn*-2/*sn*-3 molecular species refer to the dominant fatty acid combinations at the *sn*-1, *sn*-2 and *sn*-3 position of the glycerol backbone as detected in MS spectra (see Materials and Methods). * indicates the major molecular species reported in *Chlamydomonas reinhardtii.*

**carbon:double bonds**	***sn*-1/*sn*-2/*sn*-3**	
52:3	18:1/16:1/18:1	9.32%
**52:6***	16:4/18:1/18:1 + (16:3/18:1/18:2 + 16:3/18:0/18:3)	8.47%
52:4	18:1/18:2/16:1 + 18:2/18:2/16:0 +18:3/18:1/16:0	8.14%
52:2	18:1/16:0/18:1	7.90%
52:5	18/18/16 combinations	7.50%
52:7	16:4/18:1/18:2 + (16:3/18:1/18:3 + 16:3/18:2/18:2)	6.47%
54:3	18:1/18:1/18:1	5.26%
52:8	16:4/18:3/18:1 + 16:4/18:2/18:2	5.00%
50:5	16:4/18:1/16:0 + 16:0/16:3/18:2	4.64%
54:5	18:1/18:1/18:3 + 18:2/18:2/18:1 + 20:0/18:1/16:4	4.42%
54:4	18:1/18:2/18:1	4.39%
50:6	16:4/18:2/16:0	3.60%
54:6	18:1/18:2/18:3 + 18:1/18:1/18:4 + 18:2/18:2/18:2 + 16:4/20:0/18:2	3.33%
52:9	16:4/18:1/18:4 + 16:4/18:2/18:3	3.08%
50:4	10:0/18:1/16:3	2.63%
48:9	16:4/18:0/18:4	2.19%
54:8	18:3/18:2/18:2 + 18:3/18:3/18:1 + 18:4/18:2/18:1 + 16:4/20:0/18:3	2.03%
50:7	16:4/18:3/16:0	1.99%
50:3	18:2/14:0/18:1 + (16:0/18:3/16:0)	1.90%
54:2	18:1/18:1/18:0	1.87%
50:2	18:1/16:0/16:0	1.08%
