# Supplementary material for: Adaptive traits of cysts of the snow alga Sanguina nivaloides unveiled by 3D subcellular imaging
Source: Nat Commun. 2023 Nov 18;14:7500. doi: 10.1038/s41467-023-43030-7 (PMC10657455; doi:10.1038/s41467-023-43030-7)
Supplement: Supplementary file 4 — Description of Additional Supplementary Files [file 41467_2023_43030_MOESM4_ESM.pdf]

Description of Additional Supplementary Files:

Supplementary Movie 1: Microscopic water streams at the surface of ice grains

Supplementary Movie 2: Red snow X-ray tomography

Supplementary Movie 3: FIB-SEM stack of chemically-fixed Sanguina nivaloides cysts

Supplementary Movie 4: Overview of chemically fixed red snow sample

Supplementary Movie 5: FIB-SEM stack of cryo-fixed Sanguina nivaloides cysts

Supplementary Movie 6: Overview of cryo-fixed red snow sample

Supplementary Movie 7: Sanguina and bacteria 3D segmentation

Supplementary Movie 8: Cryo-fixed Sanguina nivaloides cell 2 used for volumetric study

Supplementary Movie 9: Sanguina nivaloides lipid droplet nano-structure

Supplementary Movie 10: Whole cell architecture of Sanguina nivaloides cyst highlighting mitochondria proximity to chloroplastic starch
